# Network Biology Approaches to Achieve Precision Medicine in Inflammatory Bowel Disease

**DOI:** 10.3389/fgene.2021.760501

**Published:** 2021-10-21

**Authors:** John P Thomas, Dezso Modos, Tamas Korcsmaros, Johanne Brooks-Warburton

**Affiliations:** ^1^ Earlham Institute, Norwich, United Kingdom; ^2^ Quadram Institute Bioscience, Norwich, United Kingdom; ^3^ Department of Gastroenterology, Norfolk and Norwich University Hospital, Norwich, United Kingdom; ^4^ Department of Gastroenterology, Lister Hospital, Stevenage, United Kingdom; ^5^ Department of Clinical, Pharmaceutical and Biological Sciences, University of Hertfordshire, Hatfield, United Kingdom

**Keywords:** inflammatory bowel disease, network biology, protein-protein interaction network, gene coexpression network, multilayered network, precision medicine, gene regulatory network, metabolic network

## Abstract

Inflammatory bowel disease (IBD) is a chronic immune-mediated condition arising due to complex interactions between multiple genetic and environmental factors. Despite recent advances, the pathogenesis of the condition is not fully understood and patients still experience suboptimal clinical outcomes. Over the past few years, investigators are increasingly capturing multi-omics data from patient cohorts to better characterise the disease. However, reaching clinically translatable endpoints from these complex multi-omics datasets is an arduous task. Network biology, a branch of systems biology that utilises mathematical graph theory to represent, integrate and analyse biological data through networks, will be key to addressing this challenge. In this narrative review, we provide an overview of various types of network biology approaches that have been utilised in IBD including protein-protein interaction networks, metabolic networks, gene regulatory networks and gene co-expression networks. We also include examples of multi-layered networks that have combined various network types to gain deeper insights into IBD pathogenesis. Finally, we discuss the need to incorporate other data sources including metabolomic, histopathological, and high-quality clinical meta-data. Together with more robust network data integration and analysis frameworks, such efforts have the potential to realise the key goal of precision medicine in IBD.

## Introduction

Inflammatory bowel disease (IBD), comprising Ulcerative Colitis (UC) and Crohn’s disease (CD), is a chronic, immune-mediated inflammatory disorder which primarily involves the gastrointestinal tract (Lennard-Jones, 1989; Baumgart and Carding, 2007). It causes significant morbidity and affects almost seven million people worldwide. The prevalence is forecasted to rise steeply in the decades ahead, particularly in newly industrialised countries (GBD 2017 Inflammatory Bowel Disease Collaborators, 2020). IBD arises due to a dysregulated immune response secondary to complex interactions between multiple genetic risk factors, a “dysbiotic” gut microbiota, and environmental factors (Xavier and Podolsky, 2007; Cader and Kaser, 2013). However, the precise mechanistic pathways interlinking these various facets of IBD pathogenesis are still largely unknown (Cader and Kaser, 2013). In addition, despite recent advances in medical management including the use of biologic and small molecule therapies, a significant proportion of patients who wish to avoid surgery fail to achieve sustained clinical remission (Cosnes et al., 2011). This highlights the need for novel, effective therapeutic strategies in IBD.

Unlike rare, and well-defined monogenic disorders (e.g., cystic fibrosis) which occur due to mutations within a single gene, complex diseases such as IBD arise due to interactions between numerous genetic variants and environmental factors. These interactions occur across several layers that transcend the ecologic, genetic, epigenetic, protein and cellular levels, and work collectively to manifest the disease phenotype. Consequently, IBD demonstrates significant heterogeneity across the population i.e., patients may have varying environmental exposures and express different genetic variants which result in the activation of varying pathogenic pathways. Hence, a one-size-fits-all approach to therapy, as is currently practised, may explain the suboptimal clinical outcomes seen in IBD.

As a result, precision medicine has been identified as a key strategy for improving clinical outcomes in IBD (Denson et al., 2019; Verstockt et al., 2021). Precision medicine aims to harness the biological characteristics of individual patients to tailor the right therapy to the right patient at the right time (Whitcomb, 2019). This would require an understanding of the function of individual biological components and also the holistic effects of their multifactorial interactions to stratify patients (Green et al., 2017; Sudhakar et al., 2021). Whilst still in its infancy, an early example of this approach in IBD is the PROFILE study. In this trial, researchers are utilising a transcriptomic signature of peripheral blood CD8^+^ T lymphocytes as a biomarker to separate CD patients into two subgroups according to predicted disease course to guide therapeutic strategy i.e. “step up” vs “top down” therapy (Noor et al., 2020). This transcriptomic signature was found to be effective for prognostication through an earlier non-interventional study (Biasci et al., 2019). It is anticipated that multi-omics approaches may be even more robust for directing precision therapies in IBD and other complex disorders (Olivera et al., 2019; Borg-Bartolo et al., 2020). In this effort, over the past decade, researchers across the world have begun profiling the transcriptomics, epigenetics, metabolomics, and proteomics data of large patient cohorts. For IBD, a number of biorepositories have become established such as the IBD BioResource in the United Kingdom (Parkes and IBD BioResource Investigators, 2019), the 1000IBD project in the Netherlands (Spekhorst et al., 2017), and the IBD Multiomics Data project in the USA (Imhann et al., 2019). However, this exponential increase in the availability of molecular data harnessed through “omics” technologies has created one of the biggest challenges we face in biology in the 21st century i.e., what is the best way to make meaningful sense of this data to ultimately improve clinical outcomes in individual patients?

Systems biology and artificial intelligence are two complementary fields that are driving novel computational biology approaches to address this challenge. Systems biology is an interdisciplinary field that allows the systematic study of complex interactions in biological systems using a holistic approach (Ahn et al., 2006; Breitling, 2010). Artificial intelligence, on the other hand, is a domain within computer science which leverages computer systems to perform tasks that normally require human intelligence including problem-solving and decision-making (Meskó and Görög, 2020). Machine learning and deep learning, which are subdomains of artificial intelligence, offer a number of potential solutions to tackle this problem. We have previously reviewed these approaches in depth in the context of IBD (Seyed Tabib et al., 2020). In this narrative review, however, we will focus on the utility of network biology, a subfield of systems biology, to facilitate precision medicine in IBD.

Network biology is one of the fundamental tenets of systems biology, which involves using mathematical graph theory to represent, integrate, and analyse biological processes and data through networks (Pavlopoulos et al., 2011). Depending on the type of data, various biological networks can be produced, such as protein-protein interaction networks, gene regulatory networks, and metabolic networks (Vidal et al., 2011). Using network-based methods as an integration and modelling tool, important molecular interactions can be unravelled. When applied to individual patients, personalised network analysis can lead to the identification of new disease subtypes and therapeutic targets, which facilitates novel drug discovery, biomarker discovery, and drug repurposing as has been seen in cancer (Módos et al., 2017). Hence, network biology can be a valuable tool for analysing multi-omics patient data to achieve the key goal of precision medicine in IBD and other complex disorders (Korcsmaros et al., 2017).

Although in its nascent stages, in this narrative review we will highlight a variety of innovative network biology approaches that are bringing the promise of precision medicine closer to a translational reality in IBD (Table 1). First, however, we will briefly discuss some of the fundamental concepts underpinning network biology.

**TABLE 1:** Characteristics of various network types discussed in this review and their main advantages and disadvantages.

Network type	Node	Edge	Required information to build the network	Pros	Cons
Protein-protein interaction networks	Proteins	Physical interactions	Measurement of the actual protein interactions e.g. using yeast two-hybrid, affinity purification mass spectrometry or small-scale binding experiments	Many different resources, based on physical interactions ensuring larger coverage	Highly incomplete, biases in network generating methods
Metabolic networks	Metabolites	Enzymes, reactions	Measured reactions of the enzymes	Most complete network type, good for systematic modelling	Need to decide what parameter to optimise
Gene regulatory networks	Transcription factors, promoters, enhancers, and target genes	Regulatory interaction	Measurement or modelling of the regulatory interactions e.g. using ChIP-seq, yeast one-hybrid, or through inference from transcriptomics	Various network building approaches to build large coverage and make it research question specific	Highly variable and state-specific, cannot infer feedback loops from transcriptomics only
Gene co-expression networks	Genes	Similarity between the expression of two genes	Gene expression measurement	Needs only transcriptomic data	Correlation does not always equal causation

## Key Principles of Network Biology

**FIGURE 1:**
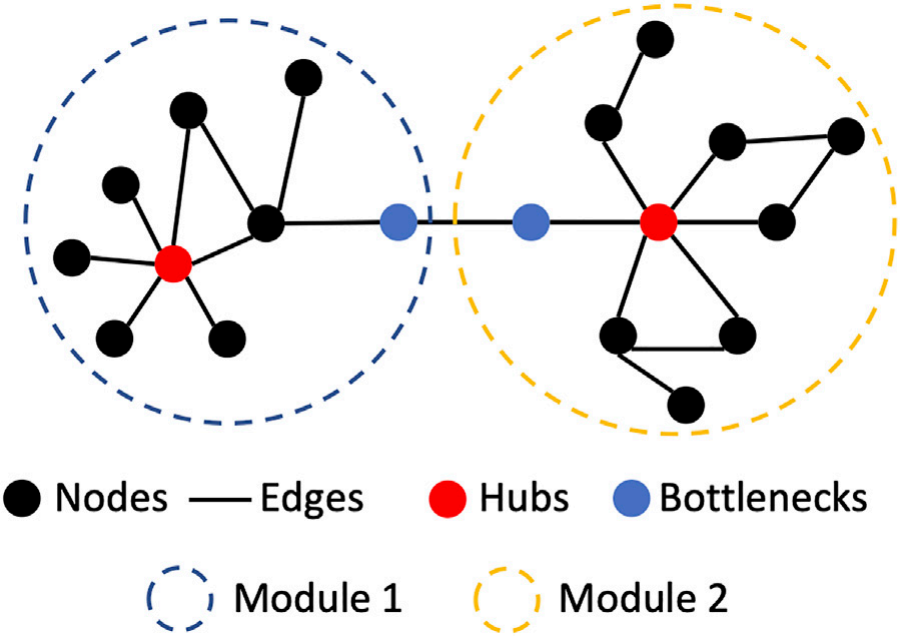
Basic network biological nomenclature and concepts. Hubs are nodes with a high number of interactions (edges). Modules are regions of the network where the nodes interact with members of the region more than with non-members. Bottlenecks are nodes which are connecting two.

A biological network is the representation of a biological system using graphs. It contains biological entities (e.g., cells, proteins or genes) and their interactions with each other (e.g., protein-protein interactions). In network biology, these are called nodes and edges, respectively (Koutrouli et al., 2020). The topology of a network (i.e., the way in which nodes and edges are arranged within a network) can be evaluated to better understand a biological system (Figure 1). In biological networks, the topology is usually scale-free i.e., the degree distribution of nodes follows a power law, unlike random networks (Barabási and Oltvai, 2004). This means that some nodes in a biological network may have many interactions called “hubs,” whilst other nodes may have fewer connections (Charitou et al., 2016). Furthermore, specific regions of a scale-free network can be more highly interconnected than other parts of the network. These highly connected regions of a network are called modules. Modules often correspond to specific biological functions within the overall system. Specific nodes that connect distinct modules can also be identified. These are termed “bottleneck nodes” as information needs to traverse through them for one module (or biological subtask) to communicate with another (Csermely et al., 2013).

To further analyse the topology of networks many tools have been developed. One method is to identify network motifs. Network motifs are recurring, significant patterns of interconnections within a network (Milo et al., 2002). Network motifs can provide insights into the type of signalling interactions that occur within different biological networks. For instance, feedforward loops are more common in transcriptional regulatory networks (Hong et al., 2018). Another technique to find the building blocks of a network is to identify graphlets. Graphlets are small, unique (non-isomorphic) subnetworks of a network (Przulj et al., 2004). Using graphlets, the local structure of a network can be better described (Przulj, 2007). Przulj and her colleagues have used graphlets to describe various networks including protein-protein interactions (Przulj et al., 2006) and the world trade network (Sarajlić et al., 2016).

Although it may not be possible to encapsulate all dimensions and features of a complex disease using networks, network analysis can be a valuable approach for better understanding the disease. For instance, disturbance of hubs and bottlenecks in a biological network are likely to have significant consequences on the overall functioning of system. A prime example is the mechanisms driving drug resistance in HER2-amplified breast cancer, in which hub proteins within compensatory circuits and feedback loops were identified (Lee et al., 2012). This led to novel therapeutic strategies for overcoming drug resistance and improving outcomes in these patients (Kirouac et al., 2013). With the successful implementation of network biology in breast cancer and other cancers over the past decade (Yan et al., 2016), researchers are increasingly looking to gain similar translatable insights in complex diseases such as IBD.

## Use of Network Biology Approaches in IBD

### Protein-Protein Interaction Networks

Protein-protein interaction (PPI) networks refer to networks consisting of proteins as nodes and the physical interactions between them as edges (Vidal et al., 2011) (Table 1). PPI data can be captured using several different methodologies including experimental approaches such as yeast two-hybrid assays and affinity purification coupled mass spectrometry, as well as computational predictive methods such as text-mining and machine learning approaches (Snider et al., 2015). Several resources containing PPI data are available for use including STRING (Szklarczyk et al., 2019), BioGRID (Chatr-Aryamontri et al., 2015), Bioplex (Huttlin et al., 2021), HAPPI-2 (Chen et al., 2009), HuRI (Luck et al., 2020), and IntAct (Hermjakob et al., 2004) (Table 2). PPI networks that are directed can facilitate better modelling of intra- and inter-cellular signalling. To gain information regarding the direction of PPIs, additional experimental data is often required. Several databases have performed a comprehensive manual curation of such experimental data from the literature to provide information on directed PPIs. These include SignaLink (Fazekas et al., 2013), Reactome (Jassal et al., 2020), and the community-driven WikiPathways (Kutmon et al., 2016) (Table 2). It is important to note that all network resources have drawbacks depending on the methods that were used to compile the data. Manually curated and text mining-based networks overrepresent certain genes which are hot topics of research - for instance, p53 is often a culprit. On the other hand, unbiased approaches like yeast two-hybrid or affinity purification overrepresent proteins that bind easily to other proteins like heat shock proteins. This can inadvertently implicate heat shock proteins as being associated with all diseases (Csermely et al., 2013). Hence, researchers need to be aware of the scope and bias of the network resources they use for their analysis.

**TABLE 2:** Network resources relevant to IBD research.

Name	Description	Website	Latest version (year)
STRING Szklarczyk et al. (2019)	Large PPI database with various sources and confidence scores. It contains text mining data and also other databases. It has both directed and undirected interactions	https://string-db.org/	11.5 (2021)
BioGRID Stark et al. (2006)	Genetic and protein interactions from both high and low throughput experiments	https://thebiogrid.org/	4.4.201 (2021)
BioPlex Huttlin et al. (2021)	Large affinity-purification mass spectrometry based database. It contains undirected PPI data	https://bioplex.hms.harvard.edu/	3.0 (2021)
HAPPI-2 Chen et al. (2017)	Large database collection of PPI data with confidence scores	http://discovery.informatics.uab.edu/HAPPI/	HAPPI 2.0 (2017)
IntAct Kerrien et al. (2012)	Large PPI database collection. Mostly undirected interactions	https://www.ebi.ac.uk/intact/	4.2.18 (2021)
Reactome Jassal et al. (2020)	Large reaction-centric PPI database, concentrating on signalling with well-developed toolsets. It has directed interactions	https://reactome.org/	77 (2021)
WikiPathways Martens et al. (2021)	Community curated database of signalling pathways. It has varying coverage	https://www.wikipathways.org/	September 2021 (2021)
SignaLink Fazekas et al. (2013)	Multi-layered database of signalling pathways with a manually curated core extended by regulatory data, external datasets and predictions	http://signalink.org/	3.0 (2021)
Signor Licata et al. (2020)	Manually curated signalling network	https://signor.uniroma2.it/	2.0 (2020)
CellPhoneDB Efremova et al. (2020)	Network database containing directed intercellular ligand-receptor interactions (i.e. a type of PPI network database)	https://www.cellphonedb.org/	2.1.7 (2021)
Ramilowski et al. Ramilowski et al. (2015)	Directed intercellular ligand-receptor interaction (PPI) network database developed by the FANTOM5 team	https://fantom.gsc.riken.jp/5/suppl/Ramilowski_et_al_2015/	(2015)
DoRothEA Garcia-Alonso et al. (2018)	Transcription factor (TF)-target gene (i.e. GRN) database with varying confidence levels and an easy-to-use application programming interface (API)	https://saezlab.github.io/dorothea	1.5.0 (2021)
TRRUST Han et al. (2015)	Manually curated transcription factor (TF)-target gene (i.e. GRN) database	https://www.grnpedia.org/trrust	2 (2017)
HuRI Luck et al. (2020)	References interactome of human binary protein-protein interactions captured using high throughput yeast two-hybrid assays	http://www.interactome-atlas.org/	April 2020 (2020)
ConsensusPathDB Kamburov et al. (2011)	A meta-database of binary and complex protein-protein, genetic, metabolic, signaling, gene regulatory and drug-target interactions, as well as biochemical pathways, originating from over 30 publicly available resources	http://cpdb.molgen.mpg.de/	Release 35 (2021)
OmniPath Türei et al. (2021)	One-stop solution of intracellular and intercellular interactions. It contains almost all the above mentioned databases and has a programmatically accessible application programming interface (API) both in R and *Python*	https://omnipathdb.org/	2.0 (2021)

**FIGURE 2:**
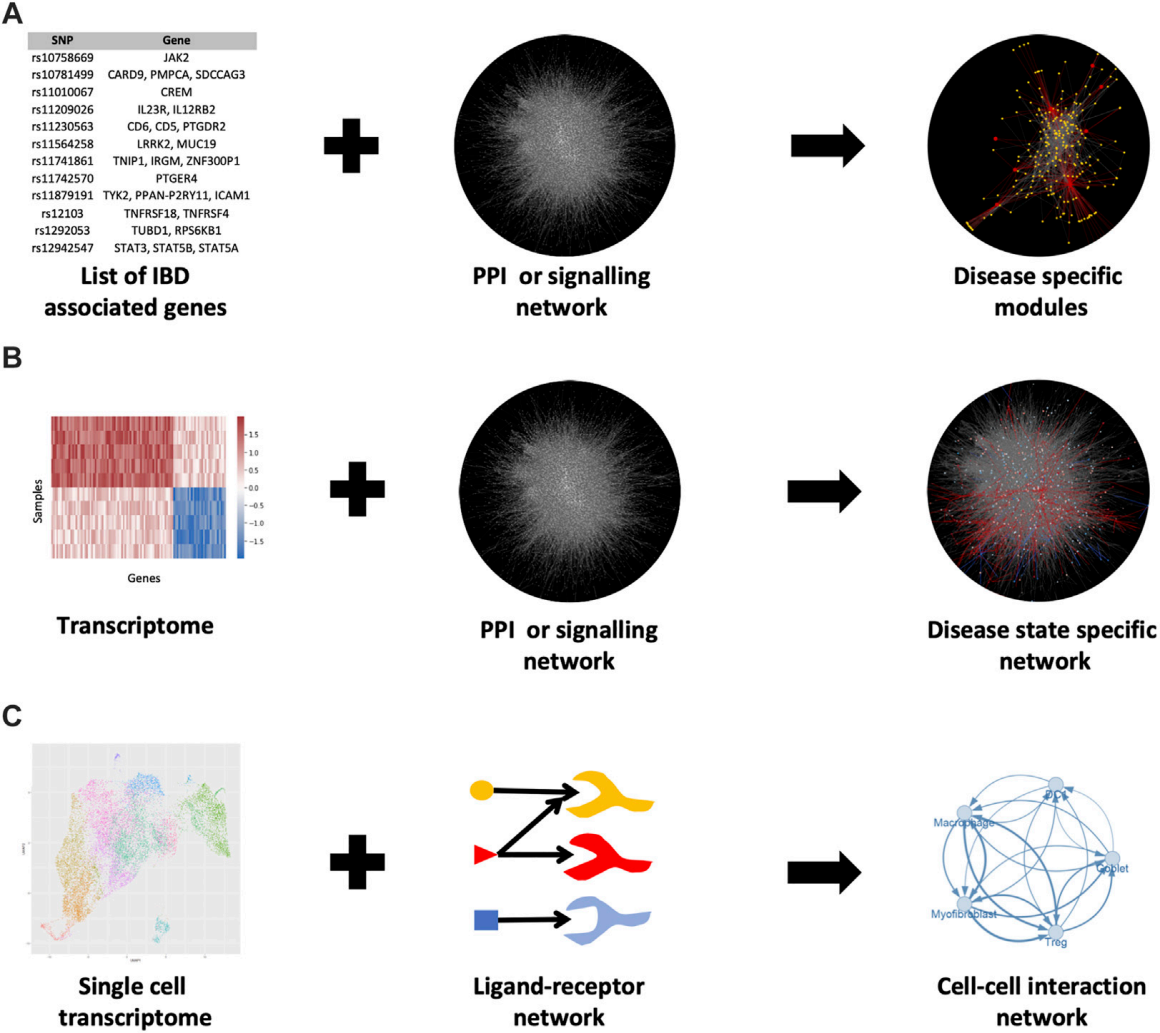
Various methods for generating PPI networks in IBD. **(A)** Known IBD-associated genes can be mapped to a PPI network and the nearby genes in the network can be associated with IBD as well (guilt by association) **(B)** Mapping a transcriptome to the PPI network can elucidate disease-specific modules in the network **(C)** Single-cell RNA-seq data combined with intercellular (ligand-receptor) communication networks can show how various cells are interacting with each other in disease or healthy states. For b) the data from (Olsen et al., 2009) was used. For c) the uniform manifold approximation and projection (UMAP) plot (a nonlinear dimensionality reduction technique for visualising high-dimensional data) was generated using data from Lukassen et al., 2020 (Lukassen et al., 2020).

By overlaying additional expression data from RNA sequencing or microarrays, PPI networks can be contextualised to specific pathological states or conditions (Figure 2). As a result, proteins are often represented by their transcripts in PPI networks. In this way, PPI networks can be used to detect novel disease-related genes, modules and signalling pathways. However, the application of transcriptomics data to build protein interactions is based on the assumption that a gene transcript accurately represents the amount of protein within the cell. This assumption is only partially true (Kosti et al., 2016).

Network propagation can also be utilised to reveal further disease-associated genes (reviewed by Cowen et al. (2017)). In short, with this approach, a set of known disease-related genes are first mapped to a PPI network and algorithms are used to detect additional proteins (or genes) that are likely to be disease-associated. Such algorithms identify additional proteins (or genes) by finding the interactor partners of the known disease-related genes using a heat propagation algorithm or a random walk approach. These methods assume that proteins (or genes) near a disease-related gene are likely to be associated with the disease as well. This is called guilt by association. Huang et al evaluated various resources that generate PPI networks to see which is the most useful for detecting disease-related genes using network propagation (Huang et al., 2018). They found that the optimal solution came from building a composite network (the parsimonious composite network or PCNet) in which interactions were supported by a minimum of two network resources.

PPI network-based approaches have been frequently used in IBD research over the past decade such as the study by Eguchi et al. (2018). In this study, the authors determined differentially expressed genes (DEGs) from transcriptomic data of IBD patients and extracted a set of known IBD genes from the DisGeNet database (Piñero et al., 2017) to construct an IBD-relevant PPI network (Figure 2B). The authors were able to identify modules within this network by using the DPClusO algorithm (Altaf-Ul-Amin et al., 2012). These IBD gene-enriched modules were used to predict novel IBD-relevant genes and pathways.

In recent years, PPI networks have also been used to generate intercellular communication networks with single-cell RNA sequencing (scRNAseq) data (Figure 2C). The method for overlaying PPI networks with scRNAseq data is dependent on the research question being asked i.e., whether the researcher is interested in studying the overall possible ligand-receptor interactions or the condition-specific changes in the strength of interactions between particular cell populations (see review by Armingol et al. (2021)). In either case, databases containing ligand-receptor interactions are required such as CellPhoneDB (Efremova et al., 2020), the FANTOM5 consortium database (Ramilowski et al., 2015) or a one-stop solution OmniPath, which we co-developed recently (Türei et al., 2021) (Table 2). OmniPath contains both ligand-receptor interactions as well as downstream intracellular signalling connections (Türei et al., 2021).

An example of such an approach using scRNAseq data in IBD is the study by Smillie et al. (2019). They obtained scRNAseq data from healthy, non-inflamed UC, and inflamed UC colonic biopsies to create PPI networks of intercellular communication (Smillie et al., 2019). The authors first identified ligand-receptor interactions within specific cell types in their scRNAseq datasets by using the FANTOM5 consortium database (Ramilowski et al., 2015). They included only ligand and receptor genes that were significantly differentially expressed between the three conditions and that were also highly-expressed cell subset markers. Using the connections between these filtered ligands and receptors they then constructed cell-cell interaction networks. Statistical analysis of this network revealed significant cell-cell interactions in the various states. In this way, the authors were able to reveal the rewiring of intercellular connections between healthy and UC states. In the healthy colonic mucosa, intercellular interactions were largely found to be occurring between cell types typically associated with colonic homeostasis such as T regulatory (Treg) cells, dendritic cell type 1 (DC1) cells, as well as CD8^+^ intraepithelial lymphocytes (IELs) and CD8^+^ IL17^+^ T cells. However, in both uninflamed and inflamed states of the UC colonic mucosa, intercellular interactions were shown to be enriched between M-like cells and inflammatory fibroblasts.

To further discern changes in intercellular communication as well as subsequent downstream intracellular signalling in UC patients, we interrogated the scRNAseq data from Smillie et al using OmniPath (Türei et al., 2021). This enabled us to build an integrated network containing both intercellular PPIs and downstream intracellular PPIs in UC patients and healthy controls. This analysis revealed significant rewiring of intercellular communication between myofibroblasts and T regulatory cells (Tregs) in UC patients in comparison to healthy individuals. These changes in intercellular interactions led to major downstream signalling differences in Tregs in UC patients, in particular the TLR4 and TLR3 pathways. These pathways regulate inflammatory cytokine expression and can decrease the abundance of Treg cells (Türei et al., 2021). These findings support the hypothesis that disruption of myofibroblast-mediated regulation of Tregs may play a key role in UC pathogenesis (Pinchuk et al., 2011).

### Metabolic Networks

**FIGURE 3:**
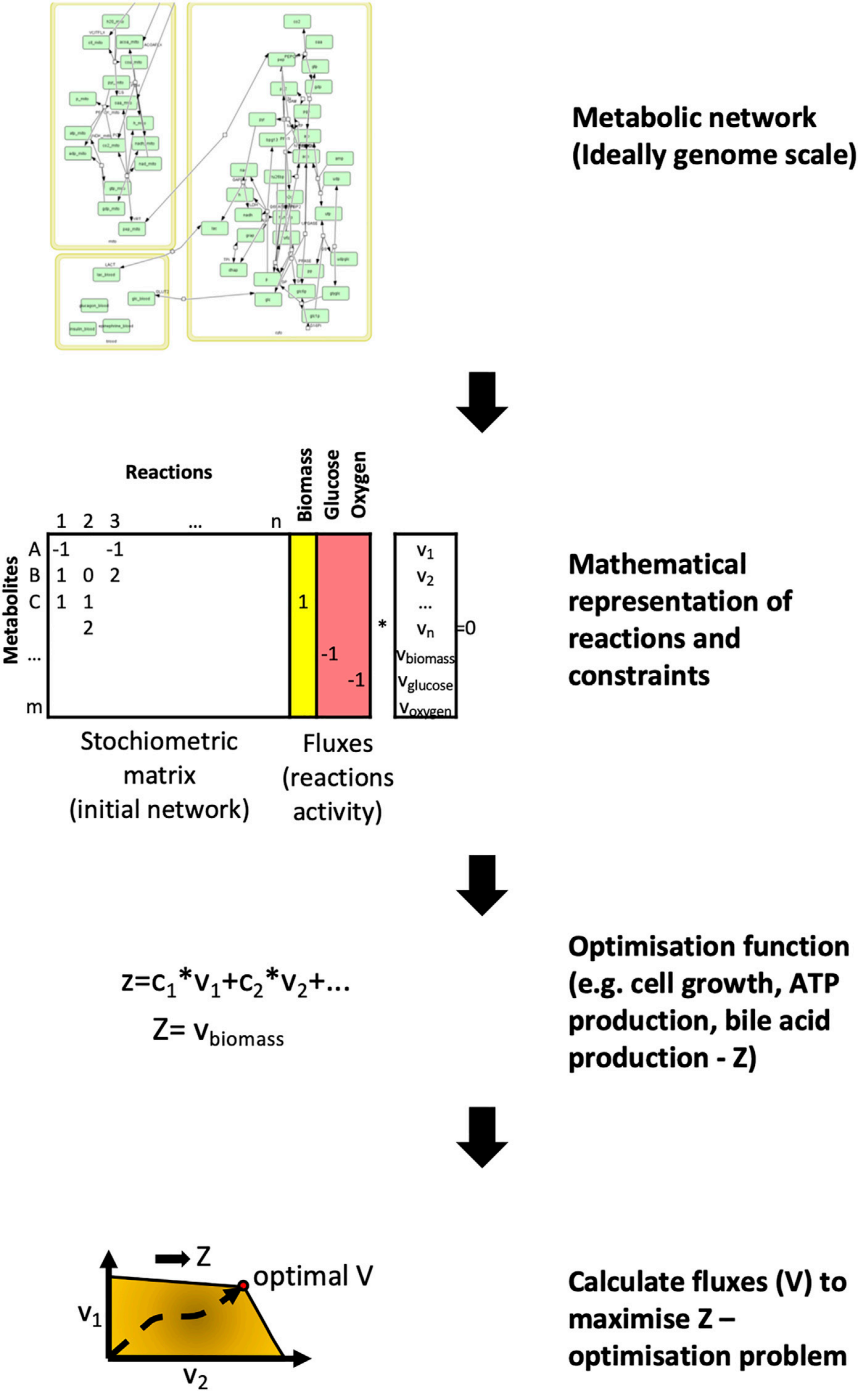
Flux balance analysis - the basics of metabolic network modelling. For metabolic networks the initial step involves collecting the metabolic reactions that form the network. These reactions are represented by a stoichiometric matrix where each reaction is represented by the nodes and metabolites by the edges. The aim of flux balance analysis is to find the optimal vector (flux) that yields the maximum output for a given metabolite or metabolites (Z) through these reactions. For illustration, the glucose metabolism was used from Köenig et al., 2012 (König et al., 2012).

In metabolic networks, nodes represent metabolites whilst edges refer to enzymes that catalyse metabolic reactions between the substrate and product metabolites (Vidal et al., 2011). The most common way of analysing a metabolic network is using flux-balance analysis, which involves calculating the flow of metabolites through the network in steady state (Orth et al., 2010; Anand et al., 2020). (Figure 3). The aim of the analysis is to find the best potential flux through the various reactions to maximise the output of a given reaction. These reactions are usually represented by cell mass or energy (ATP production). This results in an optimizable linear equation system giving back metabolic fluxes. The constraints of the model can be modified by gene expression or other experimental results. In recent years the metabolic networks of entire organisms have become available. To model the human host, the Recon2 resource provides a comprehensive global reconstruction of human metabolism (Thiele et al., 2013). For the gut microbiome, the semi-automated AGORA approach makes it possible to reconstruct the metabolism of gut microbial communities from metagenomic data (Magnúsdóttir et al., 2017). These genome-scale metabolic networks make it possible to evaluate the metabolism of the human host and gut bacterial species in the context of IBD, and discover important host-microbiome interactions (Jansma and El Aidy, 2021).

Out of all the network types reviewed here, metabolic networks have the highest completeness in terms of interactions. This makes them ideal for modelling. However, metabolomic studies are far less numerous in comparison to transcriptomics studies as RNA sequencing technologies are now far more high-throughput. In addition, a disadvantage of the standard flux balance analysis is that it needs to be optimised towards a selected metabolic reaction. When investigating IBD, the usual optimisation functions like cell growth are not relevant, so other appropriate targets need to be selected e.g., bile acid production. An alternative solution to avoid this problem is by using the metabolic network as a template and analysing it topologically (Knecht et al., 2016).

In a recent study, Heinken et al used the COBRA (Heirendt et al., 2019) genome-scale metabolic modelling software to evaluate the metabolic potential of the gut microbiome in IBD patients (Heinken et al., 2021). They found that IBD patients with dysbiosis had reduced metabolic diversity with diminished sulphur production, owing to the reduced diversity in microbial strains. In a separate study, Heinken et al also utilised flux balance analysis and genome-scale metabolic modelling to evaluate the differences in bile acid metabolism between IBD patients and healthy controls (Heinken et al., 2019). Here the optimisation function of the flux balance analysis was bile acid biotransformation. They found that one microbial species alone could not generate the whole spectrum of secondary bile acids present in the gut, but microbial pairs could generate most of these bile acids *in silico*. The network modelling also revealed that the dysbiotic microbiome of paediatric IBD patients was depleted of secondary bile acids compared to healthy children, as observed in previous studies (Duboc et al., 2013). The analysis also identified strain-specific bottlenecks that limited primary bile acid (PBA) biotransformation to secondary bile acids (SBA). Disruption of these strains may have important consequences on the inflammatory milieu in IBD, as PBAs and SBAs have been found to exert immune modulatory effects on the gut mucosa through their actions on T regulatory cells and Th17 cells (Hang et al., 2019; Sinha et al., 2020; Song et al., 2020).

An alternative approach of utilising metabolic networks to explore host metabolism in IBD was demonstrated by Knecht et al. They constructed metabolic networks by selecting enzymes which were differentially expressed between healthy controls and paediatric IBD patients from gene expression data (Knecht et al., 2016). They found that metabolic network coherence was high and varied significantly between individuals in the IBD patient cohort in comparison to healthy controls. This could have important implications for drug response in IBD patients, as metabolic networks can play a significant role in determining drug metabolism and response to treatment. Further work is needed to identify whether metabolic networks could act as a novel biomarker for determining drug response in IBD.

### Gene Regulatory Networks and Gene Co-expression Networks

A gene regulatory network (GRN) depicts the molecules that govern expression levels of genes as messenger RNA (mRNA) and proteins (Vidal et al., 2011) (Table 1). Nodes can represent transcription factor proteins, genes, *cis*- and *trans*- DNA regulatory elements, or microRNA (miRNA). Edges represent physical interactions between these molecular entities and are directed i.e. information is provided regarding whether a molecule inhibits or activates another molecule (Schlitt and Brazma, 2007). GRNs can be mapped using yeast one-hybrid (Y1H), chromatin immunoprecipitation (ChIP) approaches, ChIP-sequencing, and DNA affinity purification (Yeh et al., 2019).

**FIGURE 4:**
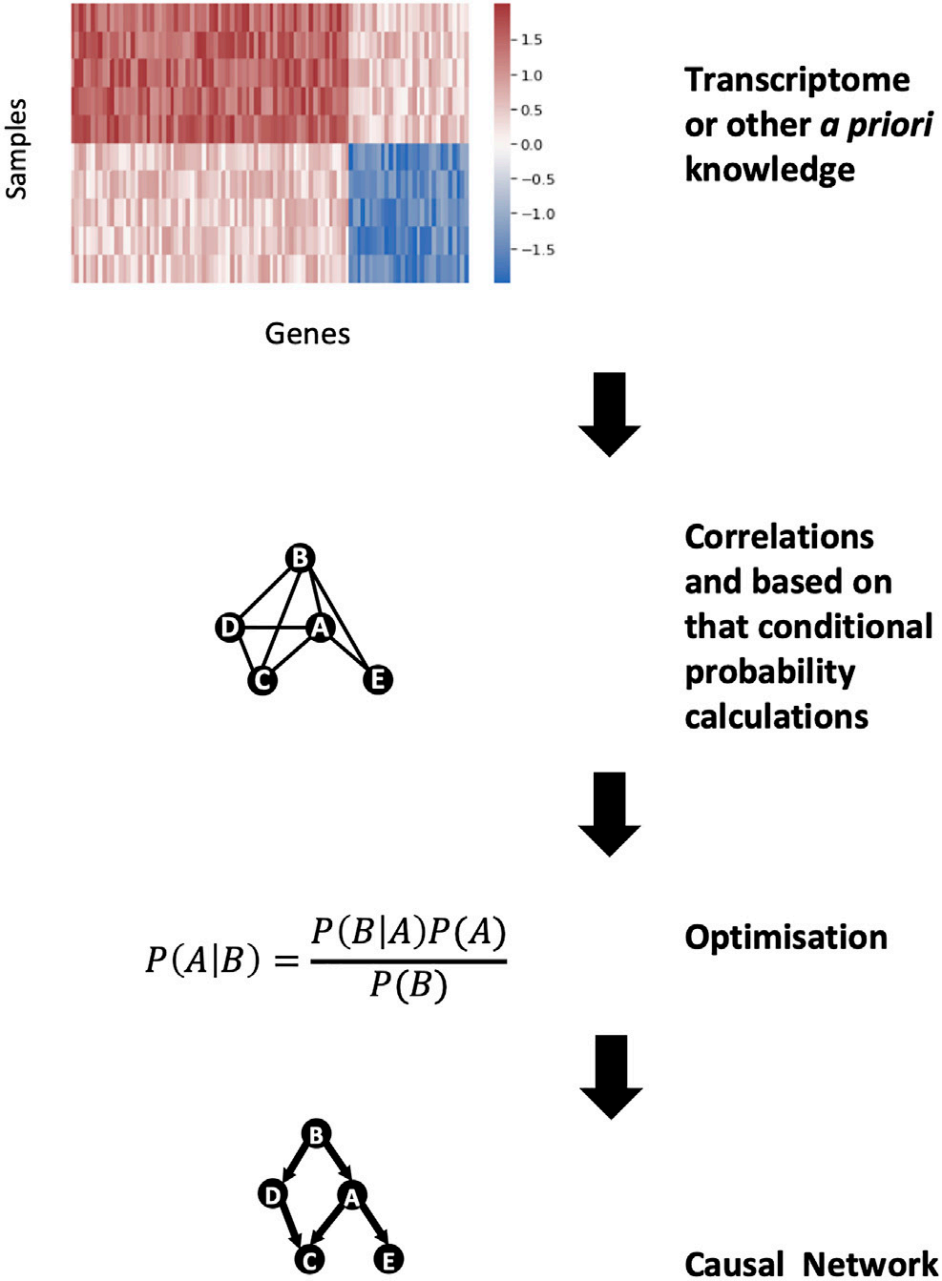
Bayesian network construction for gene regulatory networks. From the high dimension of gene expression data, the correlations between genes can be calculated. These correlations can be modelled as conditional probabilities and, using the Bayes theorem, a casual gene regulatory network can be constructed.

GRNs can be modelled with so-called Bayesian-based network inference approaches to predict the hierarchy and the directionality of the interactions in the network. Bayesian networks are founded on the Bayes theorem, which states that the probability of event A given the occurrence of another event B i.e., P (A|B), is equal to the product of the probability of event B given the occurrence of event A i.e., P(B|A) and the probability of event A i.e., P(A), divided by the probability of event B i.e., P(B) (Bayes, 1763) (Figure 4). We can predict the likelihood of event A given the occurrence of event B i.e., P (A|B), if we know how often events A and B occur and how often event B occurs given the prior occurrence of event A. The Bayes theorem can be expanded to be used with transcriptomics data, because the expression of certain genes is dependent on other genes (Friedman et al., 2000). Hence, by applying the Bayes theorem to transcriptomic data it is possible to develop a network to predict which genes are influencing the expression of other genes. As an output, a Bayesian network approach produces a hierarchical graph which reveals the most plausible causal interactions occurring between genes. However, there are two limitations with this approach. Firstly, the Bayesian graph has to be acyclic i.e., it must lack biological feedback loops. Secondly, finding the optimal Bayesian network is a computationally hard optimisation problem as a Bayesian approach results in many equally or similarly good solutions. To tackle the first issue, the research question must be properly defined i.e., research questions involving feedback loops in the biological process cannot be studied using Bayesian network approaches. The second problem can be addressed by reducing the optimisation problem to a limited search space by using predefined biologically meaningful interactions (e.g., interactions from experimentally validated sources).

**FIGURE 5:**
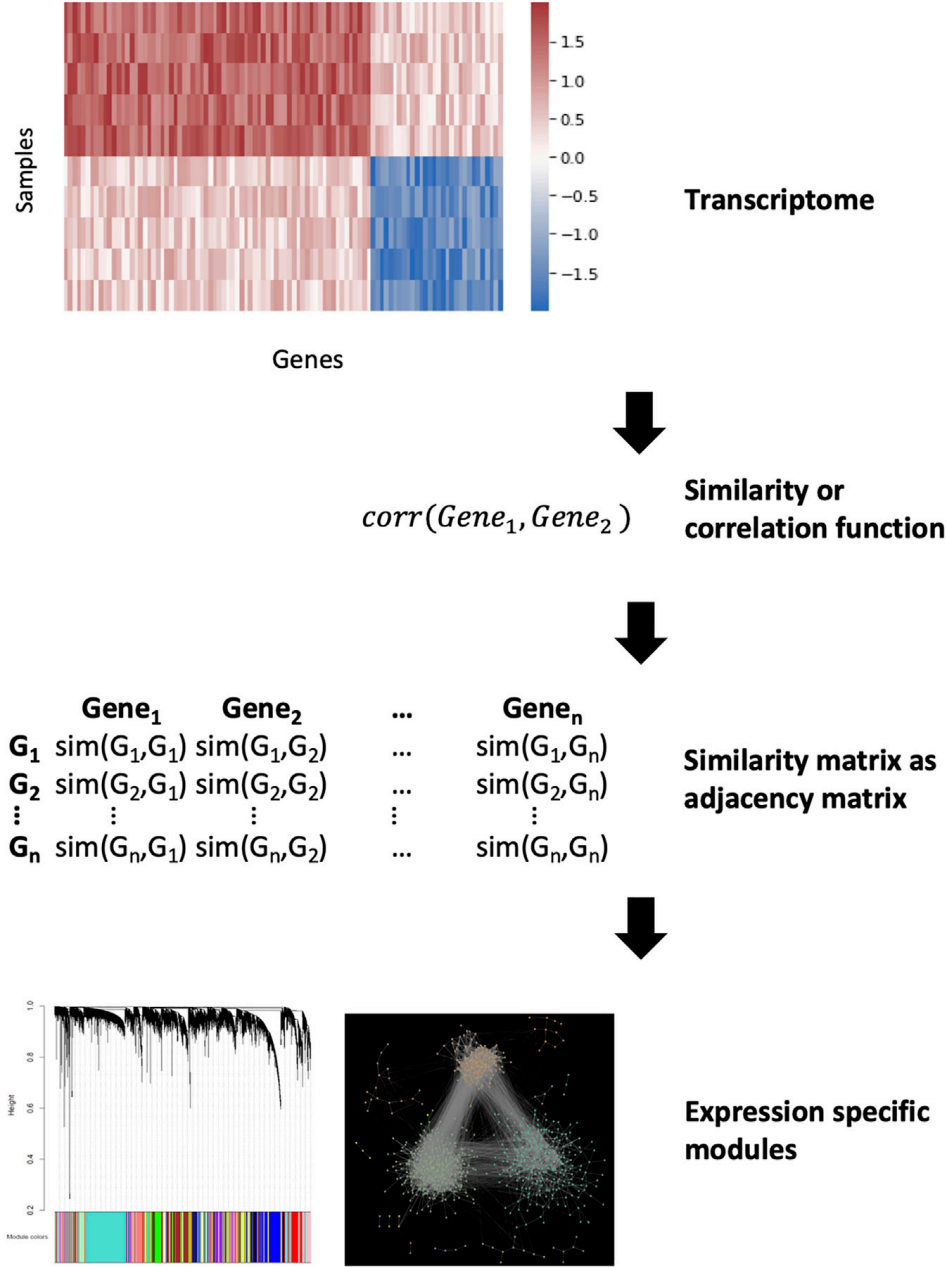
Gene co-expression network analysis. Calculating a similarity between the genes from expression data can be used as an adjacency matrix in a co-expression network. The similarity function depends on the used method but after that the most similar parts of the network can be denoted as modules.

In gene co-expression networks (GCNs), nodes represent genes and edges connect pairs of genes that are considered co-expressed based on a certain measure (Vidal et al., 2011). Unlike GRNs, edges are undirected and simply indicate a correlation in the expression of two genes, from which causality is inferred. GCNs have become a particularly popular method in recent years as they can be constructed directly from data obtained through high-throughput gene expression experiments such as microarrays or RNA-sequencing (van Dam et al., 2018). The gene co-expression can be measured using a variety of techniques (we encourage the reader to read the comprehensive review by Sonawane et al for a summary of these algorithms (Sonawane et al., 2019)). Of these, the most commonly used algorithm is the Weighted Gene Co-expression Analysis (WGCNA) (Langfelder and Horvath, 2008) (Figure 5). In essence, the WGCNA algorithm calculates the correlation between the genes. This correlation is raised on a user-defined power to filter out weak interactions resulting in a scale-free network. The adjacency matrix of this network is used for clustering to find modules which represent co-regulated biological functions. GCNs and GRNs are often used together as they complement each other. The biggest advantage of these networks is that only gene expression data is required and this can be specific for the disease in question. Furthermore, the models can be refined by adding biological constraints such as known regulatory interactions like transcription factor-target gene interactions. However, their largest drawback is the *a priori* assumption that genes which are regulated and expressed together have similar functions. This notion is not always true (Sevilla et al., 2005). GRNs are also based on the assumption that correlation implies causation.

An example of using GCNs and GRNs in IBD is the landmark study by Jostins et al. In this paper, the authors performed a meta-analysis of 15 genome-wide association studies (GWAS) of CD and/or UC, to identify 73 novel and a total of 163 IBD-associated genomic loci (Jostins et al., 2012). The authors undertook network biology analysis of this data to understand how IBD-associated loci may influence pathogenesis. They performed WGCNA of gene expression data obtained from a variety of tissues including stomach, liver, adipose tissue, and blood, and identified 211 co-expression modules. These were then screened against the IBD-associated genomic loci. They identified that IBD-associated loci were particularly enriched in a module consisting of 523 genes from omental adipose tissue obtained from morbidly obese patients (i.e. the “IBD-enriched module”). Jostins *et al* also used a Bayesian network inference method to create a GRN for IBD. To do this, they combined both genotype and gene expression data to infer a direction in terms of causality for the effect of single nucleotide polymorphisms (SNPs) on the identified gene expression. The overlap between this network and the genes in the IBD-enriched module revealed a sub-network of genes that were highly expressed in bone marrow-derived macrophages. Thus, by using gene regulatory and gene co-expression networks, the authors were able to annotate IBD-associated GWAS loci to a particular immune cell network and infer causality.

Peters et al employed network biology approaches in three independent cohorts of IBD patients, representing distinct stages of the disease (treatment naïve paediatric patients, patients refractory to biologic therapy, and patients with advanced disease undergoing bowel resection), to identify key driver genes that regulated IBD networks (Peters et al., 2017). The authors integrated data about known IBD-associated SNPs, and expression quantitative trait loci (eQTL) and *cis*-regulatory element (CRE) data from the aforementioned IBD cohorts, to identify candidate causal IBD genes in specific immune cell types. These candidate genes from all immune cell types were then intersected with modules found within GCNs obtained from the three IBD patient cohorts. The authors then identified modules in these networks that were significantly enriched for genes within the macrophage-enriched immune network from Jostins et al. (2012). This enabled them to generate “super-immune” modules by taking the union of these modules from each cohort. By evaluating common genes of these super-modules, the authors identified a core set of IBD susceptibility genes that were conserved across all three cohorts that were also enriched for the macrophage-enriched immune network and macrophage expression. This was termed the core immune activation module (IAM). By overlaying the core IAM onto Bayesian networks constructed from gene expression data from each cohort, the authors were able to identify an IBD-specific conserved immune component (CIC) in each network. Ultimately, using this approach, the authors identified 133 key driver genes which could regulate the IBD CIC networks, five of which had not been previously associated with IBD including *DOCK2, DOK3, AIF1, GPSM3, NCKAP1L.* The expression of these genes were shown to correlate with disease duration and also were upregulated in inflamed IBD patient intestinal biopsies.

Verstockt et al demonstrated the utility of GCNs to evaluate gene dysregulation at various stages of CD (Verstockt et al., 2019). In this study, transcriptomic and miRNA data were obtained from ileal mucosal biopsies of CD patients at three different stages of their disease i.e., newly diagnosed, recurrent disease following ileal resection, and late-stage disease. The authors conducted a WGCNA on this data which revealed modules that correlated with the three disease stages. The modules positively correlating with the different stages of CD were enriched in genes relating to granulocyte adhesion, diapedesis, and fibrosis. Conversely, genes associated with cholesterol biosynthesis were enriched in the module that negatively correlated with these stages of CD. They also constructed a miRNA-target gene GRN using the Ingenuity Pathway Analysis (IPA) microRNA Target Filter tool. This revealed that dysregulated miRNAs were more abundant in newly diagnosed and late-stage CD in comparison to post-operative recurrent CD. This suggests that surgical resection of the ileum followed by ileo-colonic anastomosis may reset the gene dysregulation occurring in CD.

A recent study by Aschenbrenner et al showed how GCNs could also be used to study cytokine signalling in CD (Aschenbrenner et al., 2021). They utilised transcriptomic data of ileal biopsies from a cohort of treatment naïve paediatric CD patients and non-inflamed controls to investigate the regulation of *IL23*. IL23 is a pro-inflammatory cytokine that has been implicated in IBD pathogenesis. Genetic studies have previously identified IBD-associated SNPs affecting the *IL23R* gene (Duerr et al., 2006). Furthermore, increased production of IL23 by macrophages and dendritic cells have been detected in mouse models of colitis and IBD patients (Maloy and Kullberg, 2008). Aschenbrenner et al conducted a WGCNA to see which modules of the transcriptome from inflamed and non-inflamed tissues correlate with *IL23* expression. This analysis identified 22 gene co-expression modules. Analysis of these modules revealed that *IL23A* expression strongly correlated with the modules enriched in functions for “immune cell differentiation” and “lymphocyte differentiation.” These modules were found not to be significantly enriched in CD patients. However, an “inflammatory cytokine” module containing myeloid and stromal marker genes, proinflammatory cytokines (including OSM, IL1B, and IL6) and fibroblast activation protein, was identified that significantly correlated with IL23A expression and were also enriched in CD patients. This work supports the hypothesis that a subgroup of IBD patients may possess a pathogenic myeloid-stromal cell circuit involving OSM as identified in recent landmark studies (West et al., 2017; Smillie et al., 2019).

### Multi-Layered Network Approaches

Over the past decade, there has been an increased appetite for the capture of different types of omics data from a single sample as it is believed this could provide greater insights into disease biology. This multi-omics revolution necessitates the combination of various network modelling approaches. Multi-layered networks can be used to integrate the many facets of multi-omics data including the different time scales of biological processes (Hammoud and Kramer, 2020). In recent years, various databases have been developed such as OmniPath (Türei et al., 2021), SignaLink2 (Fazekas et al., 2013), TranscriptomeBrowser (Lepoivre et al., 2012) or ConsensusPathDB (Kamburov et al., 2011), that can be used to generate multi-layered networks to integrate multi-omics data (Santra et al., 2014).

Combining different types of networks together has unravelled important insights into IBD pathogenesis. However, such multi-layered network approaches have largely been performed on a single type of omics data so far i.e., most commonly, gene expression data. This was seen in the earlier landmark study by Jostins et al where GCNs and GRNs were used together as mentioned earlier (Jostins et al., 2012). More recently, Martin et al generated intercellular ligand-receptor networks (a type of PPI network) and GCNs from scRNAseq data obtained from ileal biopsies of patients with ileal CD (Martin et al., 2019). By applying gene co-expression analysis to the scRNAseq data, they first identified a group of cell types which strongly correlated with ileal inflammation in a subset of ileal CD patients and also lack of response to anti-TNF therapy. They termed this group the GIMATS (IgG plasma cells, inflammatory mononuclear phagocytes, activated T cells and stromal cells) module. Next they evaluated intercellular interactions communicating with the GIMATS module by using the scRNAseq data to identify experimentally validated cytokine-cytokine receptor pairs (Ramilowski et al., 2015). This revealed a distinct intercellular network driving the GIMATS module including T cells, mononuclear phagocytes, fibroblasts and endothelial cells.

Cell signalling networks are another important type of multi-layered network consisting of two components: an upstream component which is a directed PPI network containing various intracellular signaling pathways, and a downstream component which is a GRN of transcription factor-target interactions (Csermely et al., 2013). The OmniPath database is particularly useful for generating cell signalling networks as it allows the user to not only access the intracellular PPI network of a cell but also GRNs and even the extracellular ligand-receptor networks from a myriad of databases (Türei et al., 2021). Although examples of cell signalling networks have been limited in IBD thus far, recently we established a novel bioinformatic pipeline termed “iSNP”, to create a UC-specific cell signalling network from patient-derived SNP data (Brooks et al., 2019). In this approach we focused on SNPs located within non-coding regions of the genome, which represent the vast majority of SNPs associated with UC. These non-coding SNPs were annotated to transcription factor binding sites (TFBS) and miRNA-target sites (miRNA-TS) using available databases reporting transcription factor binding profiles and miRNA sequences. Protein-coding genes located within the vicinity of SNP-affected TFBS and those targeted by the SNP-affected miRNA-TS were identified using regulatory interaction data sources. In this way SNP-affected proteins were revealed. Using OmniPath, the first neighbours of these SNP-affected proteins were also pinpointed. Utilising genotyped patient data from an IBD patient cohort in East Anglia in the United Kingdom, we created individual patient-specific cell signalling networks. By applying unsupervised clustering algorithms to these patient-specific cell signalling networks, we revealed that patients clustered into four main groups and identified distinct pathogenic pathways involved in each cluster. Thus, using a novel network biology workflow involving cell signalling networks, we were able to identify distinct regulatory effects of disease-associated non-coding SNPs in subgroups of UC patients.

## Future Challenges and Potential Mitigating Strategies to Develop Network Biology Approaches for Precision Medicine

Despite the recent strides made in unravelling IBD pathogenesis using the aforementioned network biology approaches, there are several challenges that need to be overcome to achieve the goal of precision medicine in IBD (Fiocchi and Iliopoulos, 2021).

First and foremost, research efforts must focus on acquiring patient-specific data from a variety of relevant data sources that could provide a more holistic picture of the disease biology of individual patients. In the past, network biology models used only one or two dimensions of data such as PPI networks, sets of DEGs, or transcriptomic information to reconstruct biological networks (Seyed Tabib et al., 2020). However, recent breakthroughs made in cancer demonstrate that multi-layered networks which incorporate various omics data are likely to yield more powerful and translatable insights for complex diseases (Du and Elemento, 2015). There is a paucity of such approaches in IBD to date, although the aforementioned studies by Jostins et al. (2012), Martin et al. (2019) and Brooks et al. (2019) demonstrate the potential of such methods. In addition, despite the exponential increase in transcriptomics and metatranscriptomics studies in IBD in the past decade, such datasets are often limited by low patient numbers. Recently, a novel meta-analysis framework for transcriptome and metatranscriptome data in IBD has been introduced, called the IBD Transcriptome and Metatranscriptome Meta-Analysis (TaMMA) platform (Massimino et al., 2021). The TaMMA platform collates and integrates transcriptomics (and metatranscriptomics) data from multiple IBD patient cohorts using a standardised pipeline that corrects batch effects and performs differential analysis of the data. This significantly increases the sample size and statistical power for downstream analysis (Modos et al., 2021). This platform, which is available as a user-friendly, open-source web application, can maximise the utility of existing transcriptomics and metatranscriptomics datasets generated from various research centers across the world. Such meta-analysis frameworks could be a powerful way for analysing other omic layers too in the future.

**FIGURE 6:**
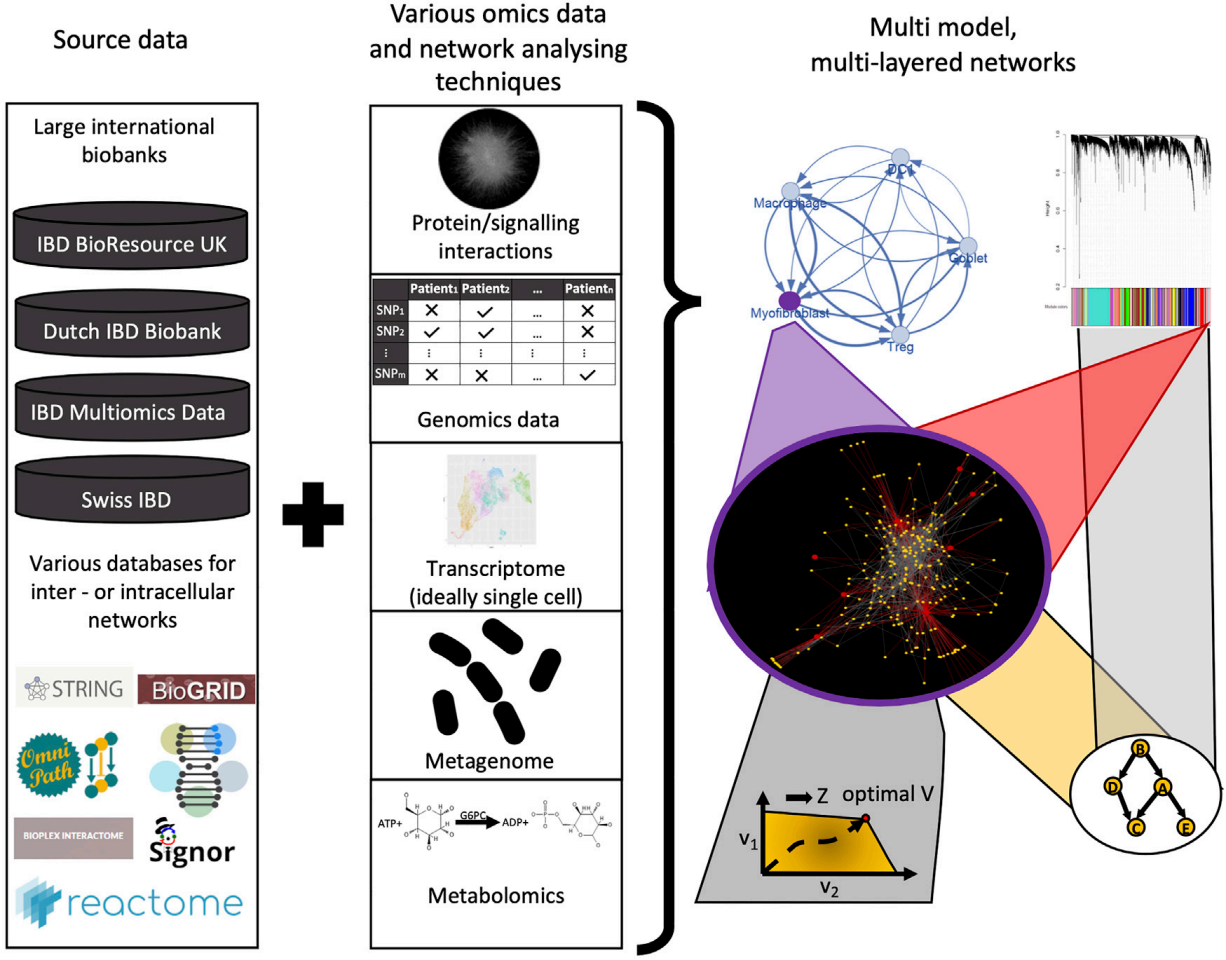
Future perspectives of using network biology and network based modeling in IBD research. From the large amount of omics datasets (genomics, transcriptomics, metabolomics, metagenomics), various interaction networks can be used to develop sophisticated network models, ideally in a multi-layered fashion. Adding granularity with patient metadata from large databases can help to validate these models and will result in better understanding of IBD pathogenesis, novel/personalised therapeutic strategies, and clinical decision-driving signatures.

In IBD, it is particularly important to consider the effects of the gut luminal microenvironment which contains bacterial cells up to 10^13^ in number and their repertoire of metabolite products on the host. However, this is an extremely complex ecosystem to model. Adding to this complexity is the dynamic nature of the gut microbiota, which can be affected by the age of the individual and environmental exposures such as diet and drugs. The development of novel genome-scale metabolic models as mentioned earlier as well as strain-specific metabolomics have potential to enhance our understanding of the IBD metabolome and the intestinal microflora (Han et al., 2021; Heinken et al., 2021). In addition to the metabolome, another data source for integration in IBD that should be strongly considered is histopathological data. The importance of integrating histopathological data for precision medicine has been clearly demonstrated in colorectal cancer (CRC) (Thomas et al., 2019). Over the past couple of decades it has been revealed that the type, density, and location of immune cells (i.e., the “immune contexture”) within CRC tissues are a better prognostic tool than the traditional Dukes staging for predicting CRC survival and recurrence (Galon et al., 2006; Fridman et al., 2012). Subsequently, transcriptomic data from CRC tissues were integrated with this histological classification, to shed light on the remarkable immunogenomic heterogeneity of CRC (Becht et al., 2016). Similar efforts to amalgamate histopathological and genomic data have thus far been scarce in IBD, but appear to be on the horizon: Friedrich et al have recently revealed distinct pathotypes in IBD that are associated with non-response to several therapies using such an approach (Friedrich et al., 2021). Furthermore, to fully realise the potential of molecular, metabolomic, and histopathological data, it is integral that they are matched with pertinent clinical metadata i.e., information of the patients’ treatment(s), age, comorbidities etc (Ahmed, 2020). This has been lacking in many previous studies (Olivera et al., 2019). However, acquiring good quality clinical metadata is challenging due to the use of paper medical records by many hospitals. Also despite the increasing use of electronic medical records (EMRs), hospitals seldom use the same EMR software resulting in interoperability issues and fragmentation of data (Warren et al., 2019). Nevertheless, artificial intelligence, including natural language processing (NLP), may help transform the extraction of clinical metadata from EMRs in the coming decades. Such big data methods can help to better understand and personalise network biology models and can also be used for validation of findings (Olivera et al., 2019; Seyed Tabib et al., 2020) (Figure 6).

Another strategy that may yield important insights into IBD disease pathogenesis is to evaluate omics data in IBD in the context of other comorbid disorders. Patients with IBD are more likely to develop other disorders with a significant immune component such as rheumatoid arthritis (RA), psoriasis, asthma and colorectal cancer (García et al., 2020). These disorders share underlying genetic risk factors and environmental exposures which can result in similarities in the immune pathways and cytokines driving inflammatory responses in these conditions (Moni and Liò, 2015). This is reflected in the fact that biologic agents targeting TNFα are effective in IBD as well as inflammatory arthritides such as RA, axial spondyloarthritis and psoriatic arthritis (Schett et al., 2021). However, thus far, there has been limited work which has evaluated multi-omics data between comorbid disease networks involving IBD. Nevertheless, bioinformatics tools have recently been generated that could be readily utilised to generate comorbidity networks from published multi-omics datasets for estimating disease comorbidity risks and patient stratification (Moni and Liò, 2015; Xiao et al., 2018).

One of the major challenges that will need to be addressed in all such approaches is how to integrate the vast amounts of multi-omics data generated from disparate sources to reveal clinically meaningful insights in IBD. Integrating genomics, transcriptomics (ideally single-cell transcriptomics), epigenomics, metabolomics, and metagenomic datasets of patients together with robust clinical meta-data and histopathological data over time will be critical for realising the goal of precision medicine in IBD (Figure 6). However, there is often a low degree of agreement between networks generated from different omics datasets, making it difficult to identify salient features that are shared between them. Therefore, more advanced data integration and analysis methods for multi-omics data are necessary.

Recently, a number of novel multi-omic data integration tools have been developed but their use has not yet penetrated the field of IBD. These include early data integration (i.e., combining all datasets into a single dataset first before developing the model) and late data integration (i.e., generating individual models from each dataset first and then finally integrating the models together) methods. Early examples of the former approach which create an aggregative layer within a multiplex network include iCluster (Shen et al., 2009), a joint latent variable model, and a similarity network fusion method by Wang et al. (2014). A weighted network fusion method has also been developed which incorporates the relative weight or importance of each layer when integrating omics layers (Angione et al., 2016). At present, one of the most common methods of omics integration is an early integration method called non-negative matrix factorisation as implemented in the MOFA package (Argelaguet et al., 2018). In short, in this method a large matrix is first constructed where the columns are the patient samples and the rows are the measurements from the various types of omics data. This large matrix is then deconvoluted into two matrices. The first matrix contains the various omics measurements as rows and factors as columns, with cells referring to the contribution of each omics measurement to a factor. Here, factors represent biological information such as signalling pathways or metabolomic circuits. The second matrix is composed of samples as columns and factors as rows, with cells referring to each factor’s value for a sample. Each factor can be traced back to the input measurements whether they are genomics, transcriptomics or metagenomic inputs. This can be used to uncover hidden interactions between various modalities of measurements. Clustering the samples based on the factors helps to reduce the noise that naturally arises when combining disparate data types. This approach was shown to identify major causes of disease heterogeneity in chronic lymphocytic leukaemia (Argelaguet et al., 2018). Late data integration methods have also revealed important insights into disease pathogenesis. An example is the COSMOS tool, in which multiple networks generated from different omics data are integrated using causal reasoning (Dugourd et al., 2021). In this paper, the investigators demonstrated the capability of COSMOS to integrate PPI, GRN and two different metabolic networks from transcriptomics, phosphoproteomics, and metabolomics data in clear cell renal cell carcinoma. Similar non-matrix-based omics methods were used in bacteria such as the MORA approach, which integrates various layers of omics data (transcriptomic, proteomics, metabolomics, genomics) to identify the affected pathways (Bardozzo et al., 2018). This method used mutual synchronisation of binarised omics measurements rather than a matrix deconvolution approach to identify affected pathways.

Recently, Malod-Dognin et al described the application of a novel multi-omics data integration and analysis framework in four different cancer types based on a machine learning technique called non-negative matrix tri-factorisation (NMTF) (Malod-Dognin et al., 2019). For each cancer type, using this approach they were able to integrate three different types of omics tissue-specific molecular interaction networks (i.e., PPI, GCN and gene interaction network) into a single, unified representation of a tissue-specific cell, which they termed “iCell.” The NMTF algorithm is an intermediate data integration method i.e., it integrates the information from the various models (networks) and source data (gene expression) giving back valuable information such as clustering of genes or local rewiring of various genes in many networks. It uses an already filtered network for this purpose. The method deconvolutes the adjacency matrices of networks into three smaller matrices per network. Two of the matrices are the same in the various networks and they are transpose of each other that capture sample-specific features, whilst the third matrix displays network-specific features. This was shown to overcome the problems associated with early data integration and late data integration approaches that have been used previously, leading to more accurate predictions. To further analyse these integrated networks, they then utilised graphlets as a more sensitive method for evaluating network topology (Przulj, 2007; Yaveroğlu et al., 2014). The distribution of graphlets can act as a fingerprint for a network, allowing comparisons to be made between networks (Sarajlić et al., 2016). Overall, this innovative integrative and analytical approach was shown to better detect the functional organisation of cancer cells than from a single omics layer and it identified 63 new cancer-related genes.

## Conclusion

Network biology approaches have provided unique insights into the pathogenesis of IBD which could not have been ascertained through simple evaluation of molecular data. With the recent establishment of several large biorepositories for IBD and the advent of next-generation sequencing, we will soon be able to access high-quality omics patient data with sufficient power to tackle some of the key unanswered questions in the field. It is important that this data is complemented with other relevant data sources, especially reliable clinical metadata. Network biology will be critical for integrating the resulting multifaceted datasets to generate clinically translatable end-points. In recent years, multi-omics integrative methods have been developed and then applied successfully in the field of cancer, but have been limited in IBD and other complex diseases. Further research is required to develop more robust integrative and analytical network biology approaches for various types of omics data. Such efforts will allow us to fully harness the potential of multi-omic patient datasets to provide deeper insights into the pathogenesis of IBD and achieve the goal of precision medicine in this complex disease.

## Network Biology Glossary

**Node/vertex:** A point in a network. In biological networks it is usually a gene or protein.

**Link/edge:** The interaction between nodes. In network biology, it can be a physical interaction such as an enzymatic reaction or similarity e.g. correlation between the expression of two genes.

**Directed network:** The network’s edges are directed meaning from node “v” to node “u” is not the same as from node “u” to node “v”.

**Weighted network:** The edges of the network have weight. In network biology, weights often represent the number of interactions between cells or the strength of the interaction between proteins which can depend on the concentration or measured amount of the proteins (in case of proteomic analysis) or the amount of the genes encoding the protein (in case of transcriptomics analysis).

**Signed interaction:** It is a type of weight of the network, which informs whether the interaction is positive or negative. A negative sign means the interaction is inhibitory, whereas a positive sign means it is excitatory.

**Degree:** The number of neighbouring nodes that a particular node connects to in a network.

**Hub:** A node with high degree.

**Path:** The set of edges connecting any two nodes.

**Shortest path:** The path between two nodes which involves the least number of edges.

**Betweenness centrality:** The number of shortest paths which go through a given node or edge. It is often normalised by the number of all possible shortest paths between all nodes.

**Bottleneck:** A node with high betweenness centrality but low degree. These are critical nodes in the network because a high amount of information goes through them.

**Module/community:** A set of nodes in a network which are interacting with each other more strongly than with other nodes outside the module.

**Scale free network:** A network which has a degree (k) distribution of P(k) = k^−γ^. In practice it means that the network has a low number of high degree nodes whilst most of the nodes have a really low degree. Most biological networks closely resemble a scale free distribution.

**Adjacency matrix:** A matrix which models the network where columns and rows represent nodes and each value is an edge. If the network is undirected, then the adjacency matrix is symmetric, whereas in directed networks the adjacency matrix is asymmetric. If the network is not weighted then the values in the adjacency matrix are 1. However, in a weighted network the values are the weights.

**Gene interaction network:** A network where the edges represent whether the mutations of the genes together influence a phenotype e.g. synthetic lethality.

**Matrix deconvolution:** Representing the matrix with multiple smaller order matrices.

**Causal reasoning:** Finding the best possible path in a network where the signs match with the output of the network.

**Graphlet:** A local unique (non-isomorphic) structure of a network.

**Network motif:** An overrepresented local structure of a network (for instance a common graphlet).
